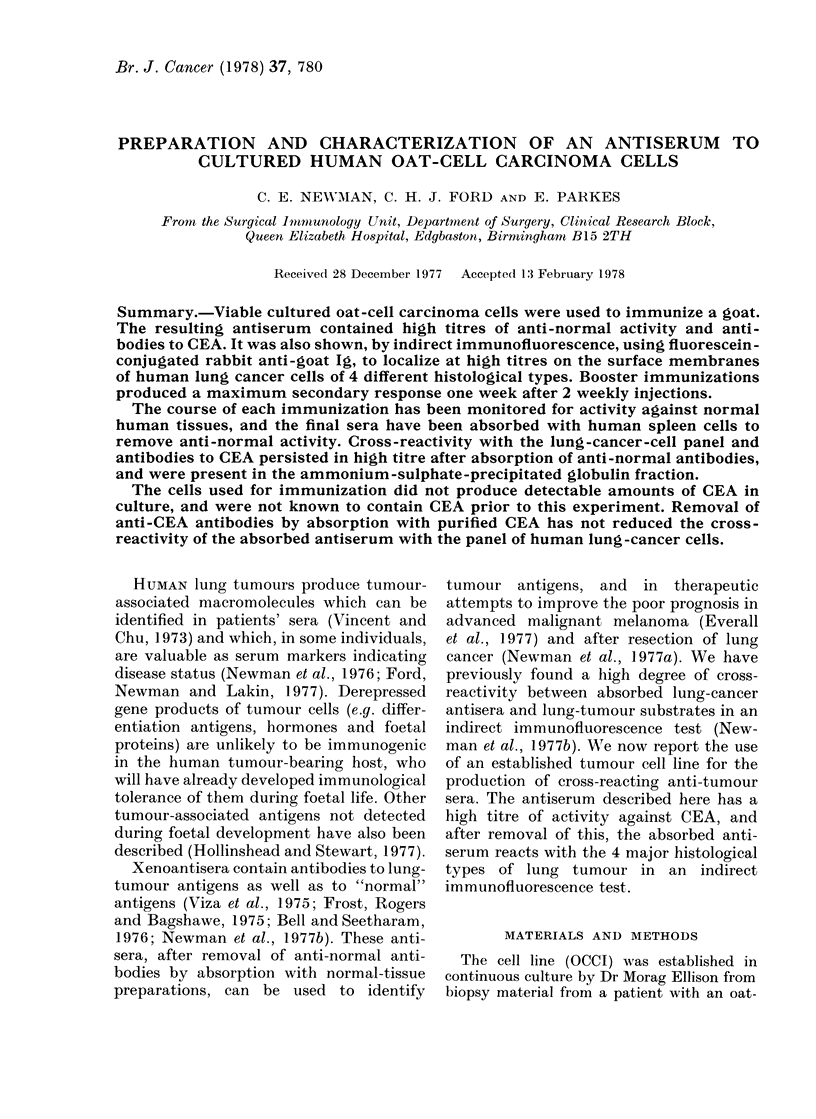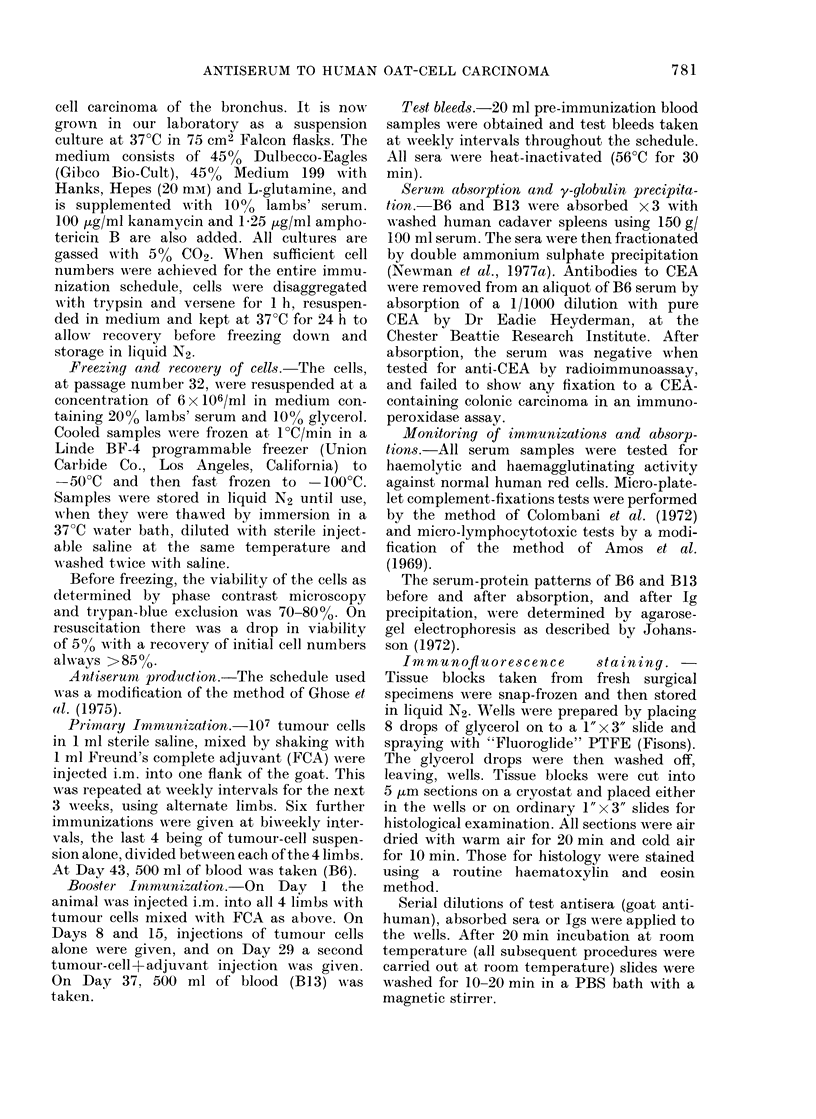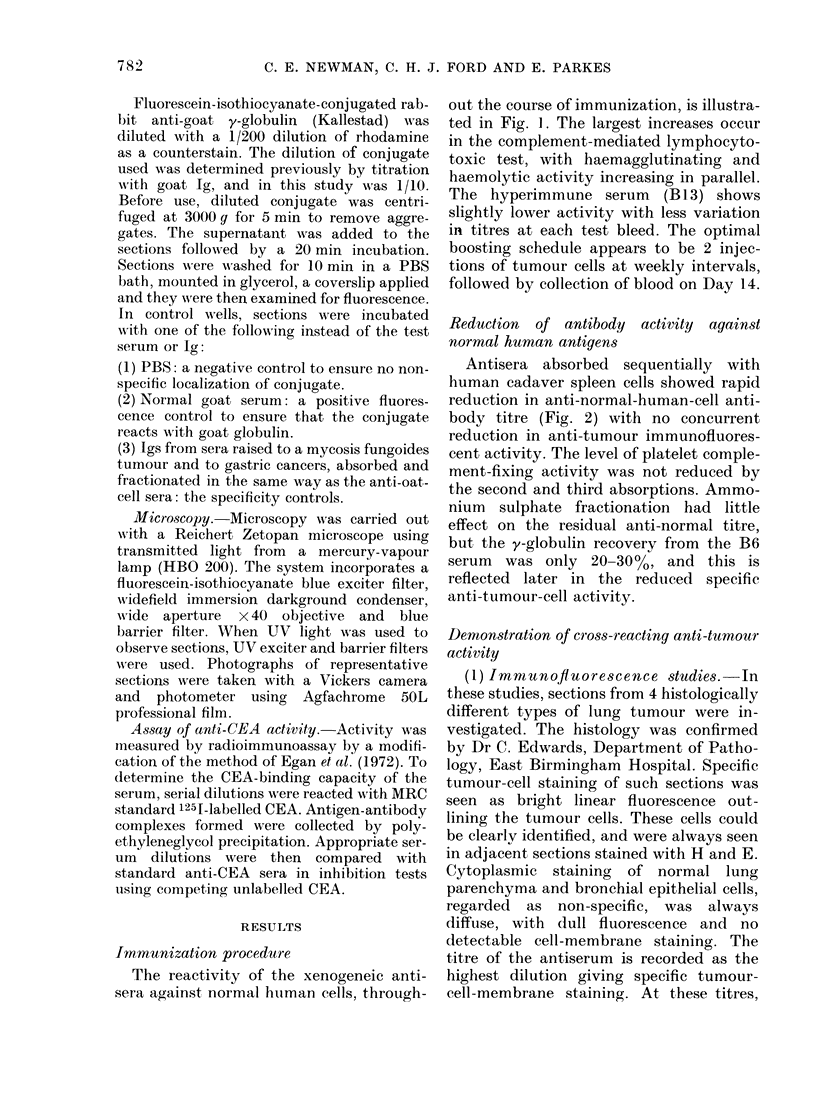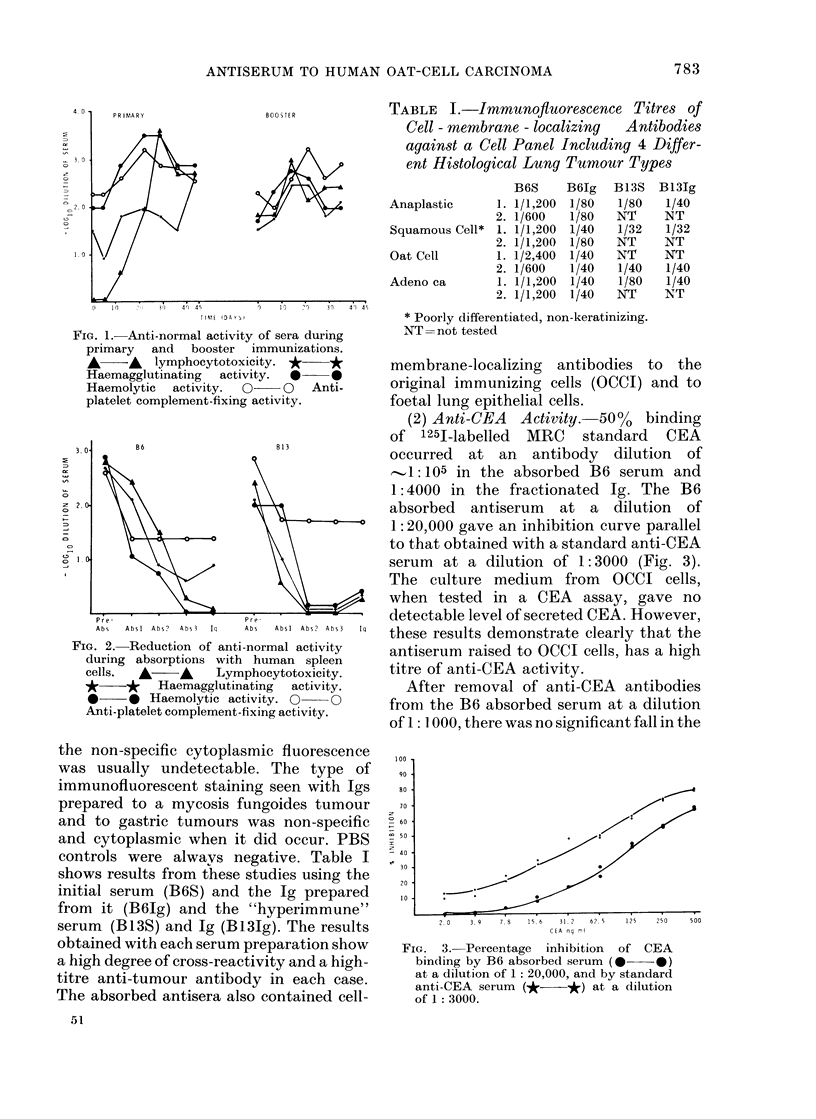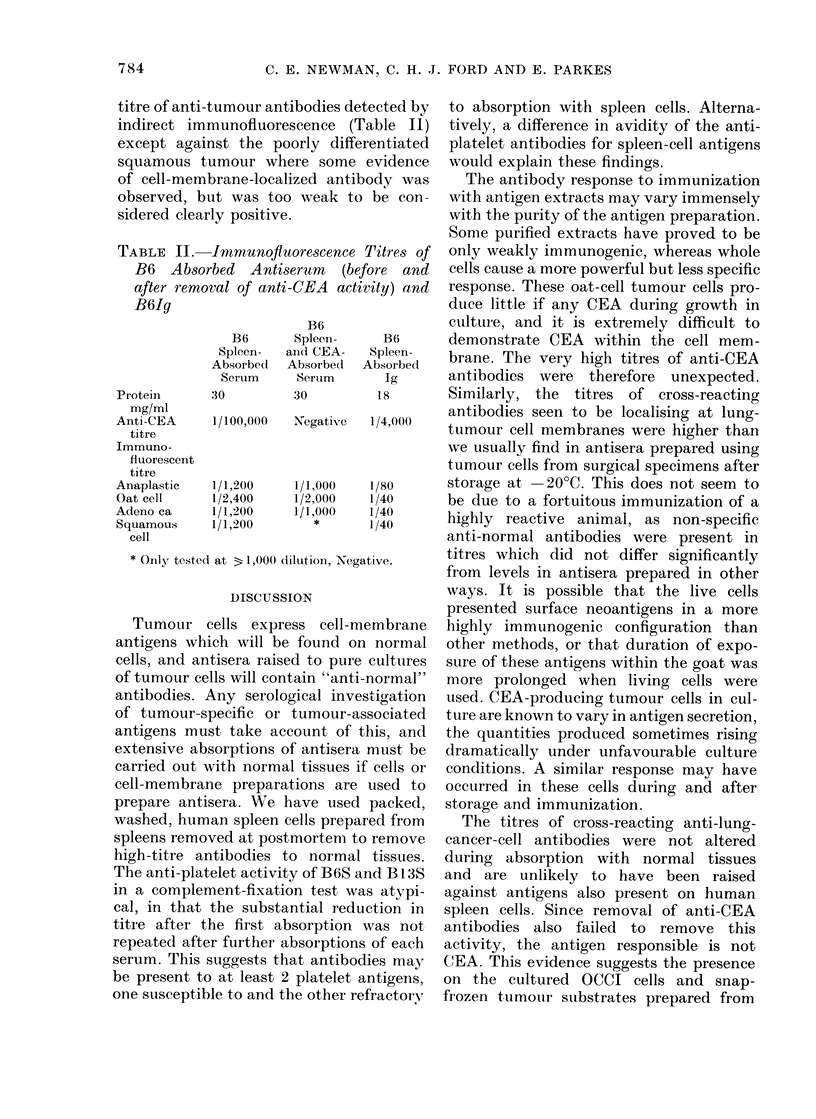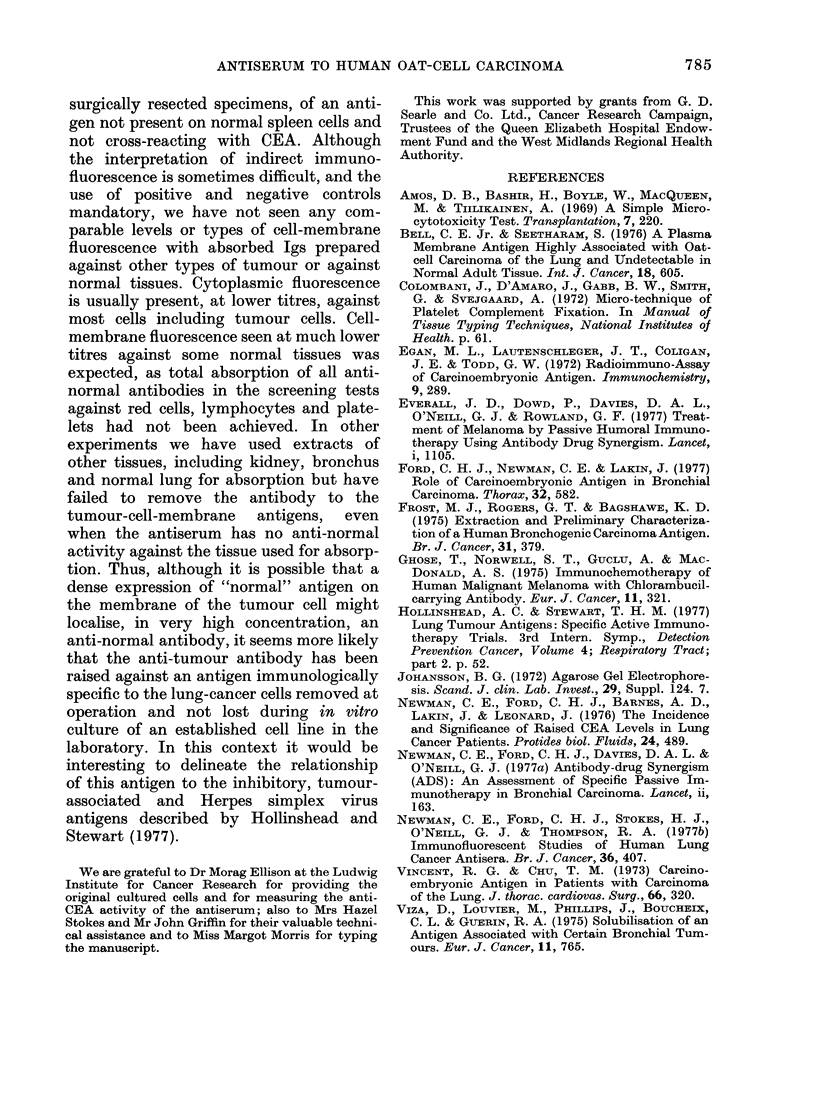# Preparation and characterization of an antiserum to cultured human oat-cell carcinoma cells.

**DOI:** 10.1038/bjc.1978.116

**Published:** 1978-05

**Authors:** C. E. Newman, C. H. Ford, E. Parkes

## Abstract

Viable cultured oat-cell carcinoma cells were used to immunize a goat. The resulting antiserum contained high titres of anti-normal activity and antibodies to CEA. It was also shown, by indirect immunofluorescence, using fluorescein-conjugated rabbit anti-goat Ig, to localize at high titres on the surface membranes of human lung cancer cells of 4 different histological types. Booster immunizations produced a maximum secondary response one week after 2 weekly injections. The course of each immunization has been monitored for activity against normal human tissues, and the final sera have been absorbed with human spleen cells to remove anti-normal activity. Cross-reactivity with the lung-cancer-cell panel and antibodies to CEA persisted in high titre after absorption of anti-normal antibodies, and were present in the ammonium-sulphate-precipitated globulin fraction. The cells used for immunization did not produce detectable amounts of CEA in culture, and were not known to contain CEA prior to this experiment. Removal of anti-CEA antibodies by absorption with purified CEA has not reduced the cross-reactivity of the absorbed antiserum with the panel of human lung-cancer cells.


*Br. J. Cancer* (1978) **37**, 780

### PREPARATION AND CHARACTERIZATION OF AN ANTISERUM TO CULTURED HUMAN OAT-CELL CARCINOMA CELLS

C. E. NEWMAN, C. H. J. FORD AND E. PARKES

*From the Surgical Immunology Unit, Department of Surgery, Clinical Research Block, Queen Elizabeth Hospital, Edgbaston, Birmingham B15 2TH*

Received 28 December 1977 Accepted 13 February 1978

**Summary.—Viable cultured oat-cell carcinoma cells were used to immunize a goat. The resulting antiserum contained high titres of anti-normal activity and antibodies to CEA. It was also shown, by indirect immunofluorescence, using fluorescein-conjugated rabbit anti-goat Ig, to localize at high titres on the surface membranes of human lung cancer cells of 4 different histological types. Booster immunizations produced a maximum secondary response one week after 2 weekly injections.**

**The course of each immunization has been monitored for activity against normal human tissues, and the final sera have been absorbed with human spleen cells to remove anti-normal activity. Cross-reactivity with the lung-cancer-cell panel and antibodies to CEA persisted in high titre after absorption of anti-normal antibodies, and were present in the ammonium-sulphate-precipitated globulin fraction.**

**The cells used for immunization did not produce detectable amounts of CEA in culture, and were not known to contain CEA prior to this experiment. Removal of anti-CEA antibodies by absorption with purified CEA has not reduced the cross-reactivity of the absorbed antiserum with the panel of human lung-cancer cells.**

HUMAN lung tumours produce tumour-associated macromolecules which can be identified in patients' sera (Vincent and Chu, 1973) and which, in some individuals, are valuable as serum markers indicating disease status (Newman *et al.*, 1976; Ford, Newman and Lakin, 1977). Derepressed gene products of tumour cells (*e.g.* differentiation antigens, hormones and foetal proteins) are unlikely to be immunogenic in the human tumour-bearing host, who will have already developed immunological tolerance of them during foetal life. Other tumour-associated antigens not detected during foetal development have also been described (Hollinshead and Stewart, 1977).

Xenoantisera contain antibodies to lung-tumour antigens as well as to "normal" antigens (Viza *et al.*, 1975; Frost, Rogers and Bagshawe, 1975; Bell and Seetharam, 1976; Newman *et al.*, 1977*b*). These antisera, after removal of anti-normal antibodies by absorption with normal-tissue preparations, can be used to identify tumour antigens, and in therapeutic attempts to improve the poor prognosis in advanced malignant melanoma (Everall *et al.*, 1977) and after resection of lung cancer (Newman *et al.*, 1977*a*). We have previously found a high degree of cross-reactivity between absorbed lung-cancer antisera and lung-tumour substrates in an indirect immunofluorescence test (Newman *et al.*, 1977*b*). We now report the use of an established tumour cell line for the production of cross-reacting anti-tumour sera. The antiserum described here has a high titre of activity against CEA, and after removal of this, the absorbed antiserum reacts with the 4 major histological types of lung tumour in an indirect immunofluorescence test.

### MATERIALS AND METHODS

The cell line (OCCI) was established in continuous culture by Dr Morag Ellison from biopsy material from a patient with an oatcell carcinoma of the bronchus. It is now grown in our laboratory as a suspension culture at 37°C in 75 $cm^2$ Falcon flasks. The medium consists of 45% Dulbecco-Eagles (Gibco Bio-Cult), 45% Medium 199 with Hanks, Hepes (20 mM) and L-glutamine, and is supplemented with 10% lambs' serum. 100 μg/ml kanamycin and 1·25 μg/ml amphotericin B are also added. All cultures are gassed with 5% $CO_2$. When sufficient cell numbers were achieved for the entire immunization schedule, cells were disaggregated with trypsin and versene for 1 h, resuspended in medium and kept at 37°C for 24 h to allow recovery before freezing down and storage in liquid $N_2$.

*Freezing and recovery of cells.*—The cells, at passage number 32, were resuspended at a concentration of $6 \times 10^6$/ml in medium containing 20% lambs' serum and 10% glycerol. Cooled samples were frozen at 1°C/min in a Linde BF-4 programmable freezer (Union Carbide Co., Los Angeles, California) to −50°C and then fast frozen to −100°C. Samples were stored in liquid $N_2$ until use, when they were thawed by immersion in a 37°C water bath, diluted with sterile injectable saline at the same temperature and washed twice with saline.

Before freezing, the viability of the cells as determined by phase contrast microscopy and trypan-blue exclusion was 70–80%. On resuscitation there was a drop in viability of 5% with a recovery of initial cell numbers always >85%.

*Antiserum production.*—The schedule used was a modification of the method of Ghose *et al.* (1975).

*Primary Immunization.*—$10^7$ tumour cells in 1 ml sterile saline, mixed by shaking with 1 ml Freund's complete adjuvant (FCA) were injected i.m. into one flank of the goat. This was repeated at weekly intervals for the next 3 weeks, using alternate limbs. Six further immunizations were given at biweekly intervals, the last 4 being of tumour-cell suspension alone, divided between each of the 4 limbs. At Day 43, 500 ml of blood was taken (B6).

*Booster Immunization.*—On Day 1 the animal was injected i.m. into all 4 limbs with tumour cells mixed with FCA as above. On Days 8 and 15, injections of tumour cells alone were given, and on Day 29 a second tumour-cell+adjuvant injection was given. On Day 37, 500 ml of blood (B13) was taken.

*Test bleeds.*—20 ml pre-immunization blood samples were obtained and test bleeds taken at weekly intervals throughout the schedule. All sera were heat-inactivated (56°C for 30 min).

*Serum absorption and γ-globulin precipitation.*—B6 and B13 were absorbed ×3 with washed human cadaver spleens using 150 g/100 ml serum. The sera were then fractionated by double ammonium sulphate precipitation (Newman *et al.*, 1977*a*). Antibodies to CEA were removed from an aliquot of B6 serum by absorption of a 1/1000 dilution with pure CEA by Dr Eadie Heyderman, at the Chester Beattie Research Institute. After absorption, the serum was negative when tested for anti-CEA by radioimmunoassay, and failed to show any fixation to a CEA-containing colonic carcinoma in an immunoperoxidase assay.

*Monitoring of immunizations and absorptions.*—All serum samples were tested for haemolytic and haemagglutinating activity against normal human red cells. Micro-platelet complement-fixations tests were performed by the method of Colombani *et al.* (1972) and micro-lymphocytotoxic tests by a modification of the method of Amos *et al.* (1969).

The serum-protein patterns of B6 and B13 before and after absorption, and after Ig precipitation, were determined by agarose-gel electrophoresis as described by Johansson (1972).

*Immunofluorescence staining.* — Tissue blocks taken from fresh surgical specimens were snap-frozen and then stored in liquid $N_2$. Wells were prepared by placing 8 drops of glycerol on to a 1″×3″ slide and spraying with "Fluoroglide" PTFE (Fisons). The glycerol drops were then washed off, leaving, wells. Tissue blocks were cut into 5 μm sections on a cryostat and placed either in the wells or on ordinary 1″×3″ slides for histological examination. All sections were air dried with warm air for 20 min and cold air for 10 min. Those for histology were stained using a routine haematoxylin and eosin method.

Serial dilutions of test antisera (goat anti-human), absorbed sera or Igs were applied to the wells. After 20 min incubation at room temperature (all subsequent procedures were carried out at room temperature) slides were washed for 10–20 min in a PBS bath with a magnetic stirrer.

Fluorescein-isothiocyanate-conjugated rabbit anti-goat γ-globulin (Kallestad) was diluted with a 1/200 dilution of rhodamine as a counterstain. The dilution of conjugate used was determined previously by titration with goat Ig, and in this study was 1/10. Before use, diluted conjugate was centrifuged at 3000 *g* for 5 min to remove aggregates. The supernatant was added to the sections followed by a 20 min incubation. Sections were washed for 10 min in a PBS bath, mounted in glycerol, a coverslip applied and they were then examined for fluorescence. In control wells, sections were incubated with one of the following instead of the test serum or Ig:

(1) PBS: a negative control to ensure no non-specific localization of conjugate.
(2) Normal goat serum: a positive fluorescence control to ensure that the conjugate reacts with goat globulin.
(3) Igs from sera raised to a mycosis fungoides tumour and to gastric cancers, absorbed and fractionated in the same way as the anti-oat-cell sera: the specificity controls.

*Microscopy.*—Microscopy was carried out with a Reichert Zetopan microscope using transmitted light from a mercury-vapour lamp (HBO 200). The system incorporates a fluorescein-isothiocyanate blue exciter filter, widefield immersion darkground condenser, wide aperture ×40 objective and blue barrier filter. When UV light was used to observe sections, UV exciter and barrier filters were used. Photographs of representative sections were taken with a Vickers camera and photometer using Agfachrome 50L professional film.

*Assay of anti-CEA activity.*—Activity was measured by radioimmunoassay by a modification of the method of Egan *et al.* (1972). To determine the CEA-binding capacity of the serum, serial dilutions were reacted with MRC standard $^{125}$I-labelled CEA. Antigen-antibody complexes formed were collected by polyethyleneglycol precipitation. Appropriate serum dilutions were then compared with standard anti-CEA sera in inhibition tests using competing unlabelled CEA.

### RESULTS

### *Immunization procedure*

The reactivity of the xenogeneic antisera against normal human cells, throughout the course of immunization, is illustrated in Fig. 1. The largest increases occur in the complement-mediated lymphocytotoxic test, with haemagglutinating and haemolytic activity increasing in parallel. The hyperimmune serum (B13) shows slightly lower activity with less variation in titres at each test bleed. The optimal boosting schedule appears to be 2 injections of tumour cells at weekly intervals, followed by collection of blood on Day 14.

### *Reduction of antibody activity against normal human antigens*

Antisera absorbed sequentially with human cadaver spleen cells showed rapid reduction in anti-normal-human-cell antibody titre (Fig. 2) with no concurrent reduction in anti-tumour immunofluorescent activity. The level of platelet complement-fixing activity was not reduced by the second and third absorptions. Ammonium sulphate fractionation had little effect on the residual anti-normal titre, but the γ-globulin recovery from the B6 serum was only 20–30%, and this is reflected later in the reduced specific anti-tumour-cell activity.

### *Demonstration of cross-reacting anti-tumour activity*

(1) *Immunofluorescence studies.*—In these studies, sections from 4 histologically different types of lung tumour were investigated. The histology was confirmed by Dr C. Edwards, Department of Pathology, East Birmingham Hospital. Specific tumour-cell staining of such sections was seen as bright linear fluorescence outlining the tumour cells. These cells could be clearly identified, and were always seen in adjacent sections stained with H and E. Cytoplasmic staining of normal lung parenchyma and bronchial epithelial cells, regarded as non-specific, was always diffuse, with dull fluorescence and no detectable cell-membrane staining. The titre of the antiserum is recorded as the highest dilution giving specific tumour-cell-membrane staining. At these titres,

FIG. 1.—Anti-normal activity of sera during primary and booster immunizations. ▲——▲ lymphocytotoxicity. ★——★ Haemagglutinating activity. ●——● Haemolytic activity. ○——○ Anti-platelet complement-fixing activity.

FIG. 2.—Reduction of anti-normal activity during absorptions with human spleen cells. ▲——▲ Lymphocytotoxicity. ★——★ Haemagglutinating activity. ●——● Haemolytic activity. ○——○ Anti-platelet complement-fixing activity.

the non-specific cytoplasmic fluorescence was usually undetectable. The type of immunofluorescent staining seen with Igs prepared to a mycosis fungoides tumour and to gastric tumours was non-specific and cytoplasmic when it did occur. PBS controls were always negative. Table I shows results from these studies using the initial serum (B6S) and the Ig prepared from it (B6Ig) and the "hyperimmune" serum (B13S) and Ig (B13Ig). The results obtained with each serum preparation show a high degree of cross-reactivity and a high-titre anti-tumour antibody in each case. The absorbed antisera also contained cell-

TABLE I.—*Immunofluorescence Titres of Cell-membrane-localizing Antibodies against a Cell Panel Including 4 Different Histological Lung Tumour Types*

| | | B6S | B6Ig | B13S | B13Ig |
|---|---|---|---|---|---|
| Anaplastic | 1. | 1/1,200 | 1/80 | 1/80 | 1/40 |
| | 2. | 1/600 | 1/80 | NT | NT |
| Squamous Cell* | 1. | 1/1,200 | 1/40 | 1/32 | 1/32 |
| | 2. | 1/1,200 | 1/80 | NT | NT |
| Oat Cell | 1. | 1/2,400 | 1/40 | NT | NT |
| | 2. | 1/600 | 1/40 | 1/40 | 1/40 |
| Adeno ca | 1. | 1/1,200 | 1/40 | 1/80 | 1/40 |
| | 2. | 1/1,200 | 1/40 | NT | NT |

* Poorly differentiated, non-keratinizing.
NT = not tested membrane-localizing antibodies to the original immunizing cells (OCCI) and to foetal lung epithelial cells.

(2) *Anti-CEA Activity.*—50% binding of $^{125}$I-labelled MRC standard CEA occurred at an antibody dilution of $\sim$1:$10^5$ in the absorbed B6 serum and 1:4000 in the fractionated Ig. The B6 absorbed antiserum at a dilution of 1:20,000 gave an inhibition curve parallel to that obtained with a standard anti-CEA serum at a dilution of 1:3000 (Fig. 3). The culture medium from OCCI cells, when tested in a CEA assay, gave no detectable level of secreted CEA. However, these results demonstrate clearly that the antiserum raised to OCCI cells, has a high titre of anti-CEA activity.

After removal of anti-CEA antibodies from the B6 absorbed serum at a dilution of 1 : 1000, there was no significant fall in the

FIG. 3.—Percentage inhibition of CEA binding by B6 absorbed serum (●——●) at a dilution of 1 : 20,000, and by standard anti-CEA serum (★——★) at a dilution of 1 : 3000.

titre of anti-tumour antibodies detected by indirect immunofluorescence (Table II) except against the poorly differentiated squamous tumour where some evidence of cell-membrane-localized antibody was observed, but was too weak to be considered clearly positive.

TABLE II.—*Immunofluorescence Titres of B6 Absorbed Antiserum (before and after removal of anti-CEA activity) and B6Ig*

| | B6 Spleen-Absorbed Serum | B6 Spleen- and CEA-Absorbed Serum | B6 Spleen-Absorbed Ig |
|---|---|---|---|
| Protein mg/ml | 30 | 30 | 18 |
| Anti-CEA titre | 1/100,000 | Negative | 1/4,000 |
| Immuno-fluorescent titre | | | |
| Anaplastic | 1/1,200 | 1/1,000 | 1/80 |
| Oat cell | 1/2,400 | 1/2,000 | 1/40 |
| Adeno ca | 1/1,200 | 1/1,000 | 1/40 |
| Squamous cell | 1/1,200 | * | 1/40 |

* Only tested at ⩾1,000 dilution, Negative.

### DISCUSSION

Tumour cells express cell-membrane antigens which will be found on normal cells, and antisera raised to pure cultures of tumour cells will contain "anti-normal" antibodies. Any serological investigation of tumour-specific or tumour-associated antigens must take account of this, and extensive absorptions of antisera must be carried out with normal tissues if cells or cell-membrane preparations are used to prepare antisera. We have used packed, washed, human spleen cells prepared from spleens removed at postmortem to remove high-titre antibodies to normal tissues. The anti-platelet activity of B6S and B13S in a complement-fixation test was atypical, in that the substantial reduction in titre after the first absorption was not repeated after further absorptions of each serum. This suggests that antibodies may be present to at least 2 platelet antigens, one susceptible to and the other refractory to absorption with spleen cells. Alternatively, a difference in avidity of the anti-platelet antibodies for spleen-cell antigens would explain these findings.

The antibody response to immunization with antigen extracts may vary immensely with the purity of the antigen preparation. Some purified extracts have proved to be only weakly immunogenic, whereas whole cells cause a more powerful but less specific response. These oat-cell tumour cells produce little if any CEA during growth in culture, and it is extremely difficult to demonstrate CEA within the cell membrane. The very high titres of anti-CEA antibodies were therefore unexpected. Similarly, the titres of cross-reacting antibodies seen to be localising at lung-tumour cell membranes were higher than we usually find in antisera prepared using tumour cells from surgical specimens after storage at −20°C. This does not seem to be due to a fortuitous immunization of a highly reactive animal, as non-specific anti-normal antibodies were present in titres which did not differ significantly from levels in antisera prepared in other ways. It is possible that the live cells presented surface neoantigens in a more highly immunogenic configuration than other methods, or that duration of exposure of these antigens within the goat was more prolonged when living cells were used. CEA-producing tumour cells in culture are known to vary in antigen secretion, the quantities produced sometimes rising dramatically under unfavourable culture conditions. A similar response may have occurred in these cells during and after storage and immunization.

The titres of cross-reacting anti-lung-cancer-cell antibodies were not altered during absorption with normal tissues and are unlikely to have been raised against antigens also present on human spleen cells. Since removal of anti-CEA antibodies also failed to remove this activity, the antigen responsible is not CEA. This evidence suggests the presence on the cultured OCCI cells and snap-frozen tumour substrates prepared from surgically resected specimens, of an antigen not present on normal spleen cells and not cross-reacting with CEA. Although the interpretation of indirect immunofluorescence is sometimes difficult, and the use of positive and negative controls mandatory, we have not seen any comparable levels or types of cell-membrane fluorescence with absorbed Igs prepared against other types of tumour or against normal tissues. Cytoplasmic fluorescence is usually present, at lower titres, against most cells including tumour cells. Cell-membrane fluorescence seen at much lower titres against some normal tissues was expected, as total absorption of all anti-normal antibodies in the screening tests against red cells, lymphocytes and platelets had not been achieved. In other experiments we have used extracts of other tissues, including kidney, bronchus and normal lung for absorption but have failed to remove the antibody to the tumour-cell-membrane antigens, even when the antiserum has no anti-normal activity against the tissue used for absorption. Thus, although it is possible that a dense expression of "normal" antigen on the membrane of the tumour cell might localise, in very high concentration, an anti-normal antibody, it seems more likely that the anti-tumour antibody has been raised against an antigen immunologically specific to the lung-cancer cells removed at operation and not lost during *in vitro* culture of an established cell line in the laboratory. In this context it would be interesting to delineate the relationship of this antigen to the inhibitory, tumour-associated and Herpes simplex virus antigens described by Hollinshead and Stewart (1977).

We are grateful to Dr Morag Ellison at the Ludwig Institute for Cancer Research for providing the original cultured cells and for measuring the anti-CEA activity of the antiserum; also to Mrs Hazel Stokes and Mr John Griffin for their valuable technical assistance and to Miss Margot Morris for typing the manuscript.

This work was supported by grants from G. D. Searle and Co. Ltd., Cancer Research Campaign, Trustees of the Queen Elizabeth Hospital Endowment Fund and the West Midlands Regional Health Authority.

### REFERENCES

AMOS, D. B., BASHIR, H., BOYLE, W., MACQUEEN, M. & TIILIKAINEN, A. (1969) A Simple Microcytotoxicity Test. *Transplantation*, **7**, 220.

BELL, C. E. Jr. & SEETHARAM, S. (1976) A Plasma Membrane Antigen Highly Associated with Oat-cell Carcinoma of the Lung and Undetectable in Normal Adult Tissue. *Int. J. Cancer*, **18**, 605.

COLOMBANI, J., D'AMARO, J., GABB, B. W., SMITH, G. & SVEJGAARD, A. (1972) Micro-technique of Platelet Complement Fixation. In *Manual of Tissue Typing Techniques, National Institutes of Health*. p. 61.

EGAN, M. L., LAUTENSCHLEGER, J. T., COLIGAN, J. E. & TODD, G. W. (1972) Radioimmuno-Assay of Carcinoembryonic Antigen. *Immunochemistry*, **9**, 289.

EVERALL, J. D., DOWD, P., DAVIES, D. A. L., O'NEILL, G. J. & ROWLAND, G. F. (1977) Treatment of Melanoma by Passive Humoral Immunotherapy Using Antibody Drug Synergism. *Lancet*, i, 1105.

FORD, C. H. J., NEWMAN, C. E. & LAKIN, J. (1977) Role of Carcinoembryonic Antigen in Bronchial Carcinoma. *Thorax*, **32**, 582.

FROST, M. J., ROGERS, G. T. & BAGSHAWE, K. D. (1975) Extraction and Preliminary Characterization of a Human Bronchogenic Carcinoma Antigen. *Br. J. Cancer*, **31**, 379.

GHOSE, T., NORWELL, S. T., GUCLU, A. & MACDONALD, A. S. (1975) Immunochemotherapy of Human Malignant Melanoma with Chlorambucil-carrying Antibody. *Eur. J. Cancer*, **11**, 321.

HOLLINSHEAD, A. C. & STEWART, T. H. M. (1977) Lung Tumour Antigens: Specific Active Immunotherapy Trials. 3rd Intern. Symp., *Detection Prevention Cancer, Volume* 4; *Respiratory Tract*; part 2. p. 52.

JOHANSSON, B. G. (1972) Agarose Gel Electrophoresis. *Scand. J. clin. Lab. Invest.*, **29**, Suppl. 124. 7.

NEWMAN, C. E., FORD, C. H. J., BARNES, A. D., LAKIN, J. & LEONARD, J. (1976) The Incidence and Significance of Raised CEA Levels in Lung Cancer Patients. *Protides biol. Fluids*, **24**, 489.

NEWMAN, C. E., FORD, C. H. J., DAVIES, D. A. L. & O'NEILL, G. J. (1977*a*) Antibody-drug Synergism (ADS): An Assessment of Specific Passive Immunotherapy in Bronchial Carcinoma. *Lancet*, ii, 163.

NEWMAN, C. E., FORD, C. H. J., STOKES, H. J., O'NEILL, G. J. & THOMPSON, R. A. (1977*b*) Immunofluorescent Studies of Human Lung Cancer Antisera. *Br. J. Cancer*, **36**, 407.

VINCENT, R. G. & CHU, T. M. (1973) Carcinoembryonic Antigen in Patients with Carcinoma of the Lung. *J. thorac. cardiovas. Surg.*, **66**, 320.

VIZA, D., LOUVIER, M., PHILLIPS, J., BOUCHEIX, C. L. & GUERIN, R. A. (1975) Solubilisation of an Antigen Associated with Certain Bronchial Tumours. *Eur. J. Cancer*, **11**, 765.